# Supplementary material for: Causal Relationship between Aging and Anorexia Nervosa: A White-Matter-Microstructure-Mediated Mendelian Randomization Analysis
Source: Biomedicines. 2024 Aug 16;12(8):1874. doi: 10.3390/biomedicines12081874 (PMC11351342; doi:10.3390/biomedicines12081874)
Supplement: Supplementary file 1 [file biomedicines-12-01874-s001.zip › Supplementary Figure/Figure S8/Leaveoneout plot of MR analysis for TL to ubm-b-2087.pdf]

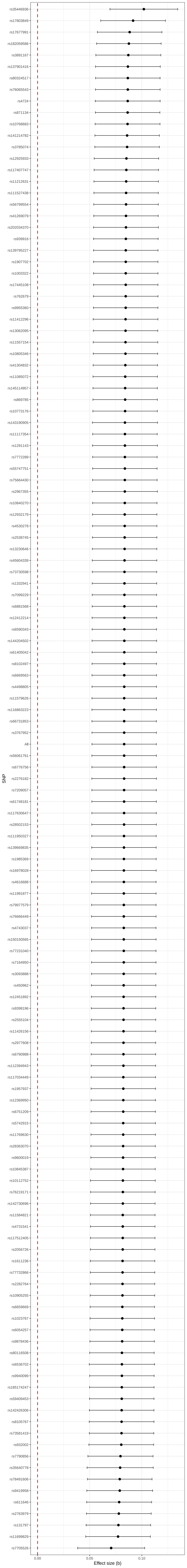

rs35446936
rs17803849
rs17677991
rs182059586
rs3891167
rs137901416
rs80324517
rs76065543
rs4724
rs871134
rs10768683
rs141214782
rs3785074
rs12925933
rs117407747
rs11212631
rs111527438
rs56799554
rs41269079
rs202034370
rs939916
rs139795227
rs1907702
rs1003322
rs17445108
rs762679
rs9955360
rs11412296
rs13062095
rs11557154
rs10805346
rs41304832
rs11085072
rs145114957
rs869785
rs10773176
rs143190905
rs11117354
rs1291143
rs7772289
rs55747751
rs75664430
rs2967355
rs10840270
rs12932179
rs4530278
rs2538745
rs13230646
rs45604339
rs73730598
rs1332941
rs7099229
rs6881568
rs12412214
rs6590343
rs144204502
rs61405042
rs8102497
rs6669563
rs4498805
rs11579626
rs116863223
rs66731853
rs3767952
A8
rs56061761
rs6776756
rs2276182
rs7209057
rs61748181
rs117630647
rs28502153
rs111950327
rs139669835
rs1985369
rs16978028
rs4616688
rs11991877
rs79977579
rs76666449
rs4743037
rs150150565
rs77231040
rs7164950
rs3093888
rs450962
rs12451892
rs9398196
rs2555104
rs11426156
rs2977608
rs6790988
rs112394943
rs117034449
rs1957937
rs12369950
rs6751209
rs5742915
rs11769630
rs28363070
rs9600019
rs10645387
rs10112752
rs76219171
rs142730696
rs11584821
rs4731541
rs117512405
rs2056726
rs1611236
rs77732866
rs2282764
rs10905255
rs6659669
rs1023767
rs6054257
rs9878436
rs80116508
rs6536702
rs9940099
rs185174247
rs59409453
rs142426306
rs8105767
rs73581419
rs932002
rs7790856
rs35640778
rs78491606
rs9419958
rs611646
rs2763979
rs131797
rs11699829
rs7705526
SNP
0.00
0.05
0.10
Effect size (b)
